# Identification of a Group’s Physiological Synchronization with Earth’s Magnetic Field

**DOI:** 10.3390/ijerph14090998

**Published:** 2017-09-01

**Authors:** Inga Timofejeva, Rollin McCraty, Mike Atkinson, Roza Joffe, Alfonsas Vainoras, Abdullah A. Alabdulgader, Minvydas Ragulskis

**Affiliations:** 1 Department of Mathematical Modelling, Kaunas University of Technology, 51368 Kaunas, Lithuania; inga.timofejeva@ktu.edu; 2 HeartMath Institute, Boulder Creek, CA 95006, USA; rollin@heartmath.org (R.M.); mike@heartmath.org (M.A.); 3 Health Research Institute, Lithuanian University of Health Sciences, 50009 Kaunas, Lithuania; joffero@gmail.com; 4 Cardiology Institute, Lithuanian University of Health Sciences, 44307 Kaunas, Lithuania; alfavain@gmail.com; 5 Research and Scientific Bio-Computing, Prince Sultan Cardiac Center, Alhasa, Hofuf 31982, Saudi Arabia; kidsecho@yahoo.com; 6 Department of Mathematical Modelling, Kaunas University of Technology, 51368 Kaunas, Lithuania; minvydas.ragulskis@ktu.lt

**Keywords:** earth’s magnetic field, geomagnetic field, heart rate variability, psychophysiology, nonlinear dynamical systems

## Abstract

A new analysis technique for the evaluation of the degree of synchronization between the physiological state of a group of people and changes in the Earth’s magnetic field based on their cardiac inter-beat intervals was developed and validated. The new analysis method was then used to identify clusters of similar synchronization patterns in a group of 20 individuals over a two-week period. The algorithm for the identification of slow wave dynamics for every person was constructed in order to determine meaningful interrelationships between the participants and the local magnetic field data. The results support the hypothesis that the slow wave rhythms in heart rate variability can synchronize with changes in local magnetic field data, and that the degree of synchronization is affected by the quality of interpersonal relationships.

## 1. Introduction

There are numerous investigations examining correlations between human health and the Earth’s magnetic field activity. Many interesting studies have shown strong influences on a number of human pathologic and behavioral states. 

It is well established that geomagnetic field line resonances and the cavity between Earth and the ionosphere generate a number of resonant frequencies that directly overlap those of the human brain, autonomic nervous system, and cardiovascular system. Of all the physiological systems studied thus far, the rhythms of the heart and the brain are most strongly associated with changes in geomagnetic conditions [1,2,3,4,5,6,7,8,9,10,11].

For example, numerous studies have demonstrated significant relationships between magnetic storms and decreased heart rate variability (HRV), the measurement of beat-to-beat changes in heart rate [12], which is suggestive of a potential mechanism linking geomagnetic activity with increased incidents of myocardial infarction and coronary disease [7,13,14,15,16,17,18,19]. In a review of the health effects of geomagnetic disturbances [20], Palmer et al. suggest these “definite conclusions”: (1) geomagnetic disturbances have a greater effect on humans at higher geomagnetic latitudes; (2) unusually high values of geomagnetic activity have an effect on cardiovascular system health; (3) unusually low values of geomagnetic activity appear to have a negative effect on health; (4) approximately 10% to 15% of people in areas studied are negatively affected by disturbed geomagnetic activity; and (5) HRV is negatively correlated with geomagnetic disturbance.

There is a wide range of magnetic waves occurring in the magnetosphere that are excited by various processes within the magnetosphere as well as the solar wind. The most common source of ultra low-frequency wave energy measured on the ground is due to the field line resonances that exhibit the largest magnetic wave amplitudes occurring in the magnetosphere [21]. The frequency of these oscillations depends on the field strength, the number of charged ions spinning around the field lines, and specifically, the length of the magnetic field line. Quasi-sinusoidal oscillations are called “Pc” (pulsations continuous), and irregular waveforms are called “Pi” (pulsations irregular). Each major type is divided into frequency ranges associated with distinct phenomena. Standing field line oscillations are typically in the Pc3 to Pc5 range, which correspond to a frequency range between 1 and 100 mHz. Oscillations classified as Pc1 and 2 are oscillations with frequencies up to 5 hertz, which are typically excited by geomagnetic substorms [22].

The ionosphere is a layer of plasma, a term that describes highly ionized gases threaded by magnetic fields, which surrounds the Earth. The charged particles in the plasma can spiral around the magnetic field lines and travel along it, creating auroras as high-energy particles flow along the field lines to the Earth’s magnetic poles. This process was described by Hannes Alfven to explain how low-frequency waves that propagate along magnetic field lines are created [23].

Standing waves in the magnetosphere involve many magnetic field lines, with lengths several times the Earth’s radius, which are excited and oscillate at their resonant frequency, similar to a plucked guitar string. Longer field lines have a lower resonant frequency, while shorter ones resonate at a higher frequency. Field lines with more or heavier particles spiraling around them tend to have lower frequencies. Changes in solar wind velocity or the polarity and orientation of the interplanetary magnetic field can have dramatic effects on the waves, as measured on the surface of the Earth [24].

Studies have shown that increased amplitudes of field line resonances can affect the cardiovascular system, most likely because their frequencies are in the same range as the primary rhythms found in the cardiovascular and autonomic nervous systems [25]. 

There has been a rapidly growing use of HRV since new devices have made obtaining the electrocardiogram (ECG) and HRV more accessible, and the understanding that HRV reflects autonomic nervous system dynamics [12] and provides an index of stress and emotions [26] and social interaction [27].

In a study conducted by Doronin et al. [28], electroencephalogram (EEG) rhythms, blood pressure, heart rate, and reaction times were compared with the low-frequency geomagnetic rhythms. They found that the oscillations in both heart and brain patterns changed simultaneously with changes in geomagnetic activity. Experiments conducted by Zenchenko et al. [29] monitored healthy individuals’ heart rates at rest and compared them with low-frequency variations between 0.5 and 3.0 mHz in the geomagnetic field. They found that in two-thirds of the experiments, there was a synchronization between the heart rhythms and the rhythms in the geomagnetic field that occurred between 4 and 30 min-long periods.

A more recent study [30] by McCraty et al. found a surprising degree of synchronization between geomagnetic activity and human nervous system function by continuous monitoring of participants HRV over a 31-day period in a group of individuals who went about their normal day-to-day lives. Overall, the study found evidence suggesting that daily autonomic nervous system activity not only responds to changes in solar and geomagnetic activity, but also is synchronized with the time-varying magnetic fields associated with geomagnetic field line resonances and Schumann resonances. More specifically, it was found that the participants exhibited a previously unidentified slow wave rhythm in their HRV, which was highly synchronized among the study participants and the time-varying magnetic field data, with a rhythm of approximately 2.5 days.

Following these findings of a significant interconnection between changes in local magnetic field activity and heart rate variability, this study examined potential relationships between human physiology (HRV), the geomagnetic field activity, and the quality of interpersonal relationships.

It has been found that individuals have widely varying levels of sensitivity to changes in the Earth’s magnetic field, and can respond in opposite ways to fluctuations in the same environmental variable [31]. In order to improve the assessment of physiological synchronization and also identify different clusters of individuals’ response patterns, we first developed and validated a new analysis approach using near-optimal chaotic attractor embedding techniques. This allowed us to identify specific patterns of synchronization between heart rate variability and local magnetic field data, and assess potential relationships between interpersonal dynamics and physiological synchronization in a group of people.

## 2. Methods and Procedures

### 2.1. Participants

During the two-week period between 26 February and 12 March 2015, a group of 20 medical students attending the Lithuanian University of Health Sciences continuously wore cardiac monitors (Bodyguard 2, Firstbeat Technologies Ltd., Jyväskylä, Finland) that gathered inter-beat intervals (IBI) from each participant. Consequently, we obtained a total of 20 IBI series, which is the time between the consecutive R wave peaks in the electrocardiogram from the 20 participants. 

### 2.2. Ethics Statement

The research met all applicable standards for the ethics of experimentation in accordance with the Declaration of Helsinki. The permit to perform biomedical investigation was granted by the Kaunas Regional Ethics Committee for Biomedical Investigations, No. BE-2-51, 23.12.2015 (copies of documents are enclosed as Supplementary Materials). Participants provided written informed consent prior to the experiment.

### 2.3. Computational Estimation of the Synchronization of a Group’s HRV Time Series with Earth’s Magnetic Field Data 

The main objective of this study was to assess the synchronization between the HRV time series of each participant and the magnetic field data. This information was then used to construct clusters of participants within the group based on the estimated synchronization between their HRV and the magnetic field. 

#### 2.3.1. Magnetic Field Data 

The local magnetic field intensity was measured using a local magnetometer located in Lithuania (Coordinates: Latitude: 55.634068 Longitude: 23.704563), which is part of the Global Coherence Monitoring Network [32]. Two magnetic field detectors (Zonge Engineering ANT-4) were positioned in the north–south and east–west axes to detect local time-varying magnetic field strengths (sensitivity 1 pT) over a wide frequency range (0.01–300 Hz) while maintaining a flat frequency response. The data acquisition infrastructure captures, then stamps, the global positioning system time, and transmits the data to the common server. Each magnetometer in the network is continuously sampled at a rate of 130 Hz. Used data can be obtained from the HeartMath Institute website [33]. 

### 2.4. Computation of the Power of Local Magnetic Field

Consider magnetic field intensity ${\left\{I_{t}\right\}}_{t=0}^{N-1}$, where t is a discrete time variable. In order to transform ${\left\{I_{t}\right\}}_{t=0}^{N-1}$ to the frequency domain the discrete Fourier transform (DFT) was used:

(1) $$f\left(\omega \right)=\overset{N-1}{\underset{t=0}{\sum }}I_{t}\cdot e^{-\frac{2\pi it\omega }{N}},\;\omega \in \mathbb{Z}$$

In order to observe changes in spectral density over time, the analysis interval was broken up into smaller sections using the discrete time short-time Fourier transform (STFT) for ${\left\{I_{t}\right\}}_{t=0}^{N-1}$:

(2) $$F\left(\theta ,\omega \right)=\overset{\infty }{\underset{t=-\infty }{\sum }}I_{t}\cdot \xi \left(t-\theta \right)e^{-it\omega },\omega \in \mathbb{Z}$$

This is essentially a partitioned form of Equation (1) using the windowing function ξ(t). A windowing function has a value close to 1 in each of a series of sliding segments of t and a value of 0 elsewhere.

The squared magnitude of the STFT F(θ,ω) results in the spectrogram of $I_{t}$, which is utilized in the subsequent analysis since the STFT provides better time resolution:

(3) $$S\left(\theta ,\omega \right)={\left|F\left(\theta ,\omega \right)\right|}^{2}$$

S(θ,ω) is typically referenced as power spectral density (PSD). Thus, the value of S(θ,ω) is interpreted as the signal power at the time interval Δθ and at the frequency range Δω.

Consider that it is required to find the local magnetic field power in the frequency range $\omega \in \left[\omega_{min};\omega_{max}\right]\;Hz$ at time interval $t_{0}$ through $t_{1}=t_{0}+\Delta \theta \;\left(s\right)$.

The power of the local magnetic field is computed using Algorithm A:

(1) Compute the spectrogram S(θ,ω) (as described previously).
(2) Crop the spectrogram $S=\min\left\{S;S_{crop}\right\}$ in order to eliminate intermittent chaotic outbreaks in the measured data due to manmade noise, lightening, etc.
(3) Apply the Gaussian median filter of dimensions 3×3 to S for the reduction of noise.
(4) Compute the signal power as $P=\overset{\omega_{max}}{\underset{\omega =\omega_{min}\;}{\sum }}\left(\frac{1}{\Delta \theta }\overset{t_{1}}{\underset{t=t_{0}}{\sum }}S\left(t,\omega \right)\right)$.

Thus, the signal power time series is the sum of the values of the spectrogram corresponding to the specified frequency and time intervals.

#### 2.4.1. Example: Computation of the Local Magnetic Field Power

**Figure 1:**
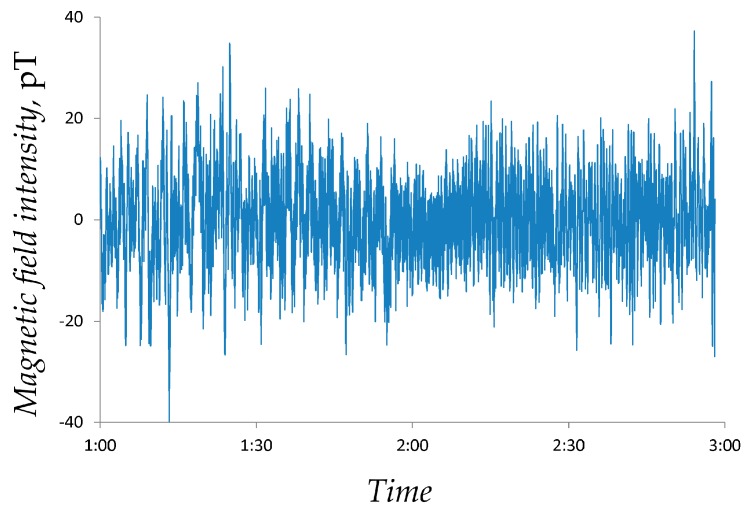
An example of the local magnetic field intensity data (measured in Lithuania during the time period between 2015/02/26 01:00:01 and 2015/02/26 03:00:01).

An example of the magnetic field intensity series is shown in Figure 1.

**Figure 2:**
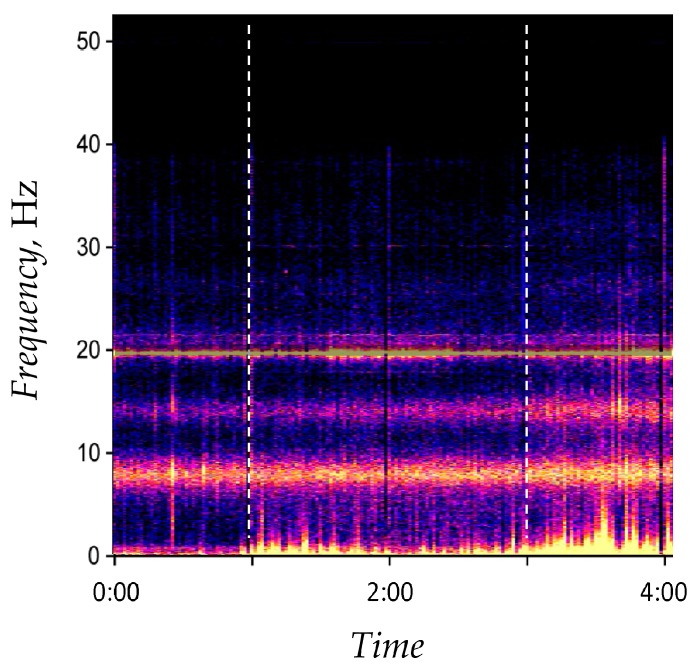
An example of the spectrogram for the magnetic field data presented in Figure 1. Frequency resolution is $\frac{1}{4096}$, Δθ=4 h, Δω=52 Hz, ω∈[0;52]Hz.

An example of the spectrogram S(θ,ω) of the magnetic field signal depicted in Figure 1 for Δθ=4 h,  ω∈[0;52]Hz is displayed in Figure 2.

In order to calculate local magnetic field power in the frequency range ω∈[0;1] Hz at time interval $t_{0}=2015/02/26\;01:00:02\;$ through $t_{1}=2015/02/26\;01:00:03$, the corresponding spectrogram is calculated as described above. The spectrogram is then cropped to S=min{S;0.25}. The signal power $P=7.7405\;(\left(\mathrm{pT}{)}^{2}/\mathrm{Hz}\right)$ is then computed from the filtered spectrogram.

### 2.5. Algorithm for the Computation of Geometrical Synchronization between Two Time Series

#### 2.5.1. Computation of the Area of an Attractor in the State Space

Let a signal $X=\left(X_{1},\ldots ,X_{n}\right)$ be a scalar time series of size n. 

It is possible to embed the time series into a 2D delay coordinate space:

$$X_{i},\;i=\bar{1,n}\to \left(X_{i},X_{i+\tau }\right),\;i=\bar{1,n-\tau },\tau \in \mathbb{N}$$

For τ=1 the following trajectory matrix is obtained:

$$\left[\begin{array}{c} \begin{array}{cc} X_{1} & X_{2}\\ X_{2} & X_{3} \end{array}\\ \begin{array}{cc} \vdots & \vdots \\ X_{n-1}\; & X_{n} \end{array} \end{array}\right],$$

where every row of the matrix corresponds to the coordinates of an embedded point in the delay coordinate space. The time lag τ can be different (τ∈ℕ). Thus, at τ=k, the trajectory matrix reads:

$$\left[\begin{array}{c} \begin{array}{cc} X_{1} & X_{k}\\ X_{2} & X_{k+1} \end{array}\\ \begin{array}{cc} \vdots & \vdots \\ X_{n-k+1}\; & X_{n} \end{array} \end{array}\right]$$

The ordered set of the embedded points is called an attractor, and the 2D plane itself is called the state space [34]. Note that the area occupied by the embedded attractor is one of the attributes characterizing the dynamics of the time series. However, the area of the attractor depends on the time lag τ used for the reconstruction of the state space. The maximal area of the embedded attractor (and the corresponding optimal time lag) is a feature that can be exploited for the description of the underlying model governing the evolution of the time series [35].

We employed a straightforward algorithm for the computation of the area of the embedded attractor based on the direct assessment of the geometric area occupied by the set of points of the trajectory matrix in the state space. The steps of Algorithm B read:

(1) Compute the center of the mass of the points comprising the attractor. Move the origin of the state space to the center of the mass.
(2) Divide the state space of the attractor into the slices with equal central angles of a circle centered on the origin. The number of slices depends on the number of points in the observation window of the time series.
(3) Set the radius of each slice to the maximal distance between a point belonging to that slice and the origin.
(4) Compute the area of the attractor $S_{\tau }$ as the sum of areas of all slices.

As noted previously, the area $S_{\tau }$ depends on τ. We consider a finite range of time lag values $\tau =1,\ldots ,\tau_{max}$; $S_{\tau }$ is calculated for each value of τ. The area of the embedded attractor is maximized in respect to τ: $\tau_{*}=\arg\left(\underset{\tau }{\max}S_{\tau }\right)$. The optimal time lag $\tau_{*}$ can be used as a scalar identifier representing the geometrical features of the analyzed data series in the corresponding observation window. The computation of the optimal time lag $\tau_{*}$ can be considered as the information reduction algorithm where a set of numbers in the original time series is mapped into a single scalar. 

#### 2.5.2. Example 1: Identification of the Optimal Time Lag

We will consider a nonlinear pendulum model with harmonic excitation as a paradigmatic chaotic oscillator in this example:

(4) $$x^{\prime \prime }+x^{\prime }+\sin\left(x\right)=b\cos\left(\omega t\right)$$

**Figure 3:**
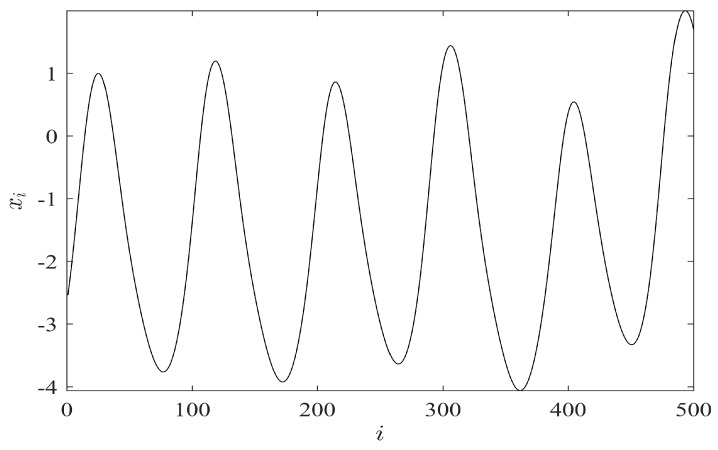
Time series $X=\left(X_{1},\ldots ,X_{n}\right)$ representing a numerical solution to Equation (4) with initial conditions $x\left(0\right)=0;\;x^{\prime }\left(0\right)=0.8$.

Equation (4) exhibits a chaotic solution at $b=2.048;\omega =\frac{2}{3}$ [36]. Figure 3 illustrates 500 data points of the chaotic solution $X=\left(X_{1},\ldots ,X_{500}\right)$. 

**Figure 4:**
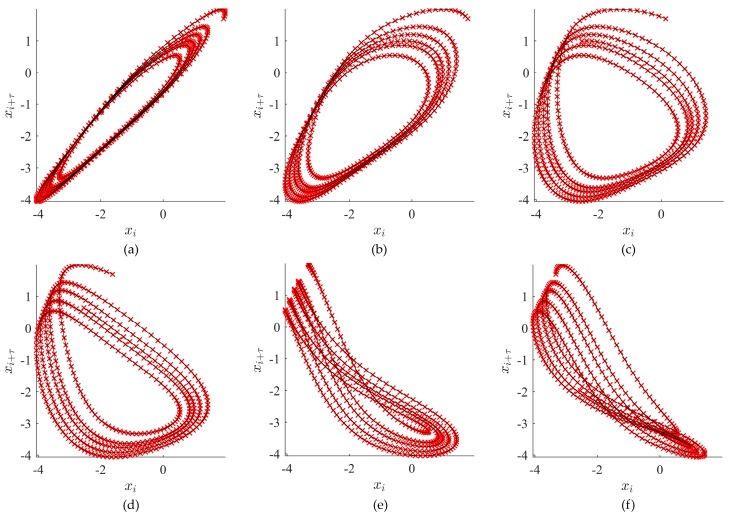
Examples of attractors for different time lag values. In (**a**) τ=4; (**b**) τ=12; (**c**) τ=23; (**d**) τ=31; (**e**) τ=43; (**f**) τ=50.

The images of embedded attractors for the time series depicted in Figure 3 using several different values of the time lag τ are presented in Figure 4.

**Figure 5:**
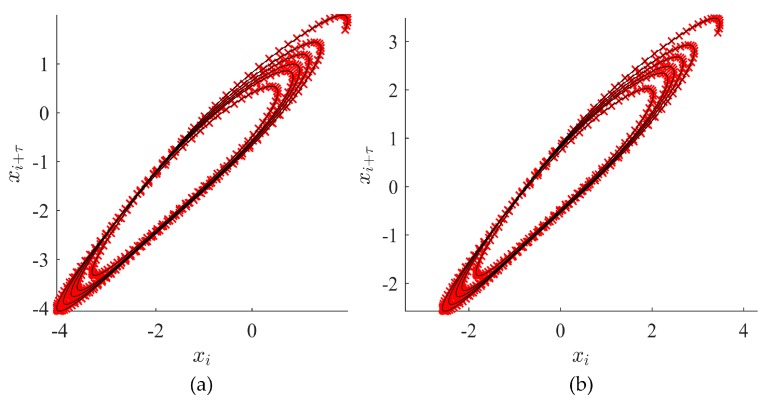
Shifting the origin to the center of mass of the attractor for τ=4: (**a**) the original attractor; (**b**) the origin shifted to the center of the mass of the attractor.

According to the algorithm presented above, the first step is shifting the origin to the center of the mass of the embedded attractor. The results of this procedure are illustrated in Figure 5. Note that this transformation does not impact the geometrical shape of the attractor.

**Figure 6:**
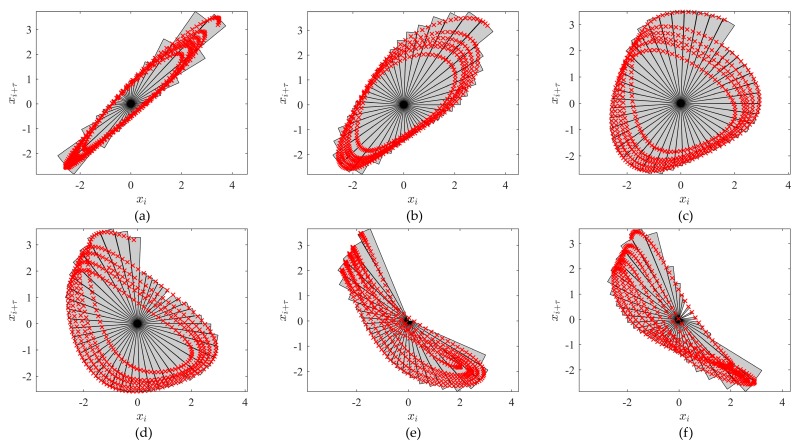
Sliced diagrams of the attractors shown in Figure 4.

We select the number of slices to be 45. The execution of steps 2 and 3 of the algorithm with the six attractors (which correspond to six distinct time lag values) shown in Figure 4 results in the corresponding sliced diagrams illustrated in Figure 6. 

**Figure 7:**
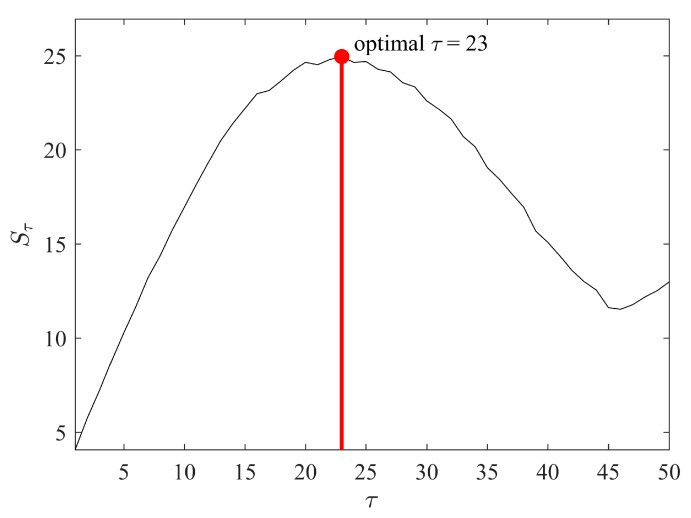
The identification of optimal time lag value $\tau_{*}$.

The maximal value of τ is set to 50, because the total number of data points in the observation window is 500 (higher values of τ would generate too short trajectory matrices). In order to compute $\tau_{*}$ in the range [1,50], the areas of embedded attractors must be computed for each value of τ. A plot representing the relationship between an area of the attractor $S_{\tau }$ and τ is presented in Figure 7. The largest area of the embedded attractor $S_{\tau }=24.9527$ is achieved at $\tau_{*}=23$.

#### 2.5.3. Construction of the Algorithm for the Estimation of the Geometrical Synchronization between Two Time Series

Let $X=\left(X_{1},\ldots ,X_{n}\right)$ and $\;Y=\left(Y_{1},\ldots ,Y_{n}\right)$ be synchronously sampled time series of size n. The following procedure for the estimation of the geometrical synchronization between those two time series. The steps of Algorithm C read:

(1) Divide signals X and Y into T observation windows of size m (m should be large enough to enable the reconstruction of a meaningful attractor in the state space):
  $$\left(X_{1},\ldots ,X_{m}\right),\left(X_{m+1},\ldots ,X_{2m}\right),\ldots ,\left(X_{n-m+1},X_{n});\;(Y_{1},\ldots ,Y_{m}\right),\left(Y_{m+1},\ldots ,Y_{2m}\right),\ldots ,\left(Y_{n-m+1},Y_{n}\right).$$
(2) Compute optimal time lags for each observation window for both time series using Algorithm B. Such computations result in two vectors of optimal time lags: $\tau_{*j}^{\left(X\right)},\;\tau_{*j}^{\left(Y\right)}\;\left(j=\bar{1,T}\right)$. This information reduction algorithm allows the identification of similarities between attractors reconstructed from different time series from the geometrical point of view. The variation of optimal time lags reconstructed for a pair of time series is used for the quantification of the generalized geometrical synchronization between those time series.
(3) Calculate the vector of absolute differences between obtained optimal time lags for each observation window: $\tau_{*j}^{\left(X,Y\right)}=\left|\tau_{*j}^{\left(X\right)}-\tau_{*j}^{\left(Y\right)}\right|\;\left(j=\bar{1,T}\right)$. The differences between the optimal time lags are used as the metric of geometrical similarity between the analyzed time series.
(4) In order to identify the slow dynamics reflecting averaged changes in absolute differences between optimal time lags for each data signal, divide the vector of absolute differences into $F=\frac{T}{h}$ segments: $\left[\tau_{*\left(h\cdot \left(i-1\right)+1\right)}^{\left(X,Y\right)},\ldots ,\tau_{*\left(h\cdot i\right)}^{\left(X,Y\right)}\right]\left(i=\bar{1,F}\right)$. The number of points h in each segment should be large enough to produce a meaningful averaging.
(5) Calculate the mean absolute difference ${\bar{\tau }}_{i}^{\left(X,Y\right)}=\frac{1}{h}\overset{h}{\underset{j=1}{\sum }}\tau_{*\left(h\cdot \left(i-1\right)+j\right)}^{\left(X,Y\right)}\;\left(i=\bar{1,F}\right)$ between optimal time lags for each segment. The obtained vector of mean absolute differences $A^{\left(X,Y\right)}=\left[{\bar{\tau }}_{1}^{\left(X,Y\right)}{\bar{\tau }}_{2}^{\left(X,Y\right)}\;\ldots \;\;\;{\bar{\tau }}_{F}^{\left(X,Y\right)}\right]$ is defined as a measure representing the geometrical synchronization between data signals X,Y.

#### 2.5.4. Computational Validation of the Geometrical Synchronization Algorithm 

To validate the analysis method, we used a system of two nonlinear pendulum models with harmonic excitation (given in Equation (4)) coupled with diffusive terms:

(5) $$x^{\prime \prime }+x^{\prime }+\sin\left(x\right)=b_{1}\cos\left(\omega t\right)+\varepsilon \left(y-x\right);\;y^{\prime \prime }+y^{\prime }+\sin\left(y\right)=b_{2}\cos\left(\omega t\right)+\varepsilon \left(x-y\right),$$

where $\omega =\frac{2}{3};b_{1}=2.048;b_{2}=2.049$. The coupling parameter ε≥0 determines the coupling strength: low values of ε correspond to low synchronization between the pendulum models, while high values lead to highly synchronized oscillations [36].

**Figure 8:**
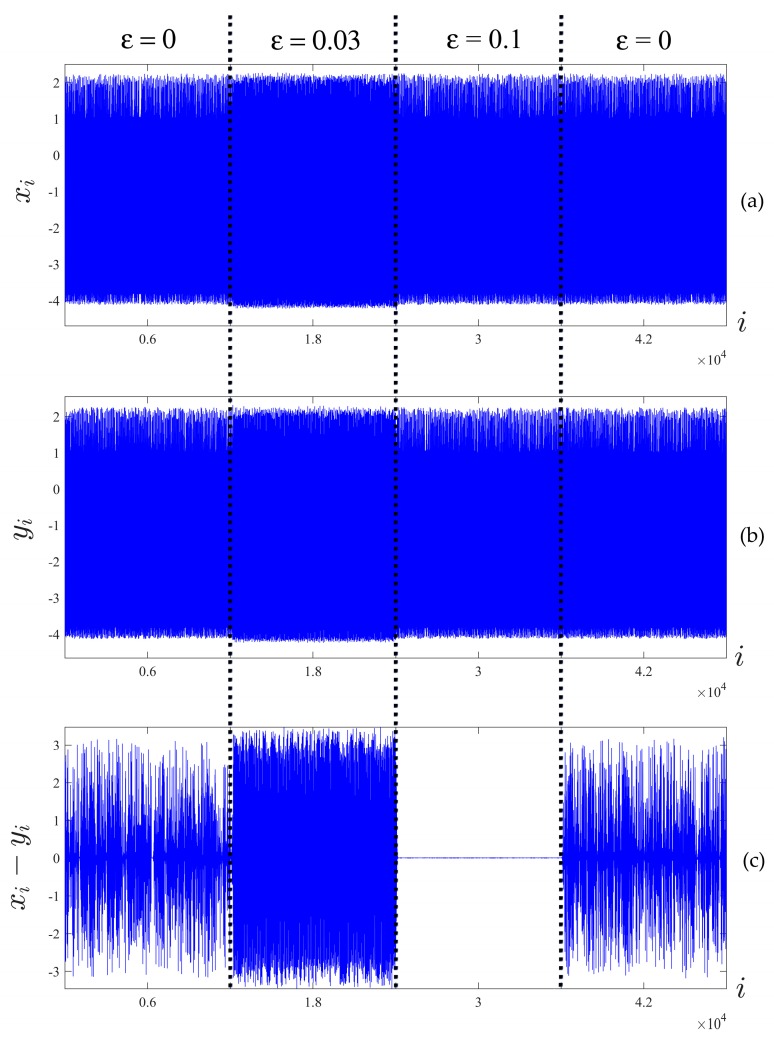
Time series X (**a**) and Y (**b**) and the difference X−Y (**c**), obtained from numerical integration of the coupled nonlinear pendulum model (Equation (5)). Dotted lines separate time intervals with different values for the coupling parameters.

Two time series $X=\left(X_{1},\ldots ,X_{n}\right),\;Y=\left(Y_{1},\ldots ,Y_{n}\right)$ of size n=48,000 are illustrated in Figure 8. Time series X and Y are constructed as follows: firstly, ε is set to zero and the equations are integrated until transient processes die down. Then, the first 12,000 data points are sampled (first quarter of Figure 8). After sampling, ε is set to 0.03 (weak diffusive coupling) and another 12,000 data points are sampled (second quarter of Figure 8). The process is repeated two more times for ε=0.1 in the third quarter, and ε=0 in the last quarter, which are shown in Figure 8.

**Figure 9:**
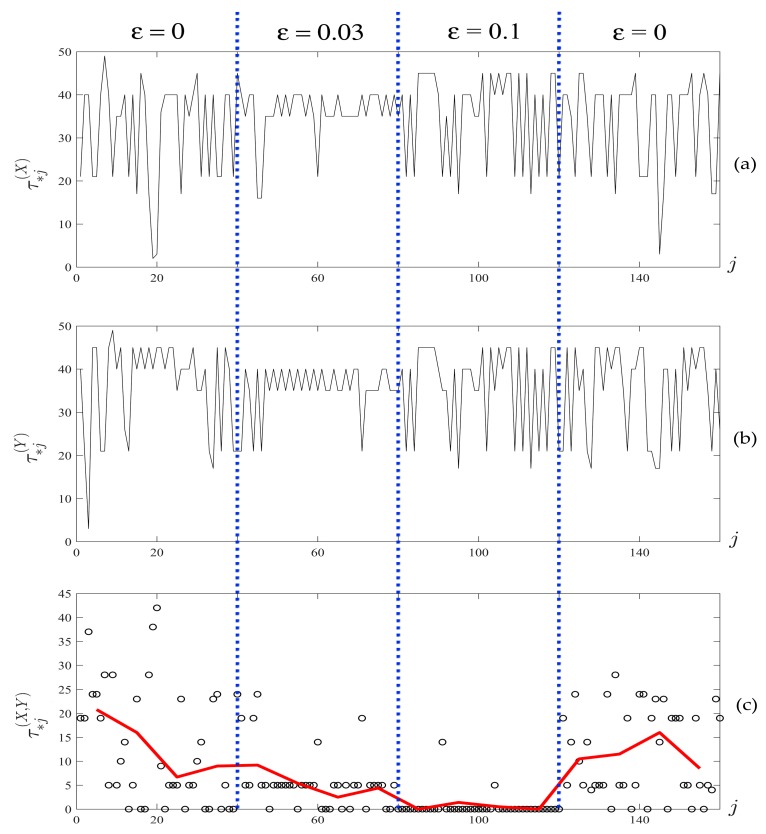
The sets of optimal time lags (**a**) $\tau_{*j}^{\left(X\right)}$ and (**b**) $\tau_{*j}^{\left(Y\right)}$ for the time series depicted in Figure 8. Circles in (**c**) denote the absolute differences $\tau_{*j}^{\left(X,Y\right)}$ between optimal time lags for X and Y. The solid red line in (**c**) corresponds to the averaged absolute differences $A^{\left(X,Y\right)}=\left[{\bar{\tau }}_{1}^{\left(X,Y\right)}{\bar{\tau }}_{2}^{\left(X,Y\right)}\;\ldots \;\;\;{\bar{\tau }}_{16}^{\left(X,Y\right)}\right]$.

The time series are divided into T=160 segments of size m=300 according to the first step of Algorithm C (300 is a sufficient number of points to reconstruct a meaningful attractor). Next, the optimal time lags for each segment of both signals were computed using Algorithm B. The set of optimal time lags $\tau_{*j}^{\left(X\right)},\;\tau_{*j}^{\left(Y\right)}\;\left(j=\bar{1160}\right)$ is presented in Figure 9a,b.

Now, absolute differences between optimal time lag vectors $\tau_{*j}^{\left(X,Y\right)}=\left|\tau_{*j}^{\left(X\right)}-\tau_{*j}^{\left(Y\right)}\right|\;\left(j=\bar{1160}\right)$ were computed. The obtained vector is divided into F=16 segments of size h=10, which results in the vector of mean absolute differences $A^{\left(X,Y\right)}=\left[{\bar{\tau }}_{1}^{\left(X,Y\right)}{\bar{\tau }}_{2}^{\left(X,Y\right)}\;\ldots \;\;\;{\bar{\tau }}_{16}^{\left(X,Y\right)}\right]$ illustrated in Figure 9c.

This procedure allows us to identify and quantify the variable of geometrical synchronization in time. The numerical values on the *y*-axis in Figure 9 can be interpreted as follows. Smaller values of ${\bar{\tau }}_{i}^{\left(X,Y\right)}$ indicate a greater magnitude of synchronization in the respective segment. If ${\bar{\tau }}_{i}^{\left(X,Y\right)}$ is equal to 0, the corresponding synchronization can be considered as absolute synchronization. Analogously, the maximal possible value of ${\bar{\tau }}_{i}^{\left(X,Y\right)}$ (equal to 49 in this Example) corresponds to absolute desynchronization. For the algorithm parameters used in this example, the cut point for desynchronization is ${\bar{\tau }}_{i}^{\left(X,Y\right)}=15$; i.e., the signals are considered poorly synchronized in the respective segment for the averaged absolute differences of optimal time lags exceeding this value. 

The chaotic oscillators given in Equation (5) are nonsynchronized in the first quarter of data points (ε=0), which results in an average absolute of differences of optimal time lags ranging from 6 to 21. When the coupling parameter is set to ε=0.03, the chaotic oscillators are weakly synchronized, which is reflected by the decreased values of $A^{\left(X,Y\right)}$. Note that this effect is not obvious, and cannot be observed by simply considering the difference X−Y (Figure 8c). Further, the coupling parameter ε=0.1 results in an almost complete synchronization, as seen from both Figure 8c and the near-zero values of $A^{\left(X,Y\right)}$ in Figure 9c. In the last quarter of data points, oscillators are allowed to evolve in the uncoupled regime at ε=0, which results in uncoupled chaotic oscillations.

### 2.6. Clusterization of Multivariate Time Series Based and Their Synchronization with a Master Time Series

Suppose a set of time series $X^{\left(k\right)}=(X_{1}^{\left(k\right)},\ldots ,X_{n}^{\left(k\right)}),\;k=\bar{1,K}$ and a master time series $M=\left(M_{1},\ldots ,M_{n}\right)$ are given. The objective of the following procedure is to compare and clusterize time series $X^{\left(k\right)}\;\left(k=\bar{1,K}\right)$ based on their synchronization in respect to the master time series M. The steps of Algorithm D read:

(1) Compute the vector of mean absolute differences $A^{\left(X^{\left(k\right)},M\right)}=\left[{\bar{\tau }}_{1}^{\left(X^{\left(k\right)},M\right)}\ldots {\bar{\tau }}_{F}^{\left(X^{\left(k\right)},M\right)}\right]$, describing the relationship between $X^{\left(k\right)}$ and M as described in Algorithm C, for each $X^{\left(k\right)},\;k=\bar{1,K}$.
(2) Calculate the Euclidean distance (the measure used to estimate the geometrical similarity of two data vectors) which represents the similarity between all K data signals, using the following formula:
  $${║A^{\left(X^{\left(i\right)},M\right)}-A^{\left(X^{\left(j\right)},M\right)}║}_{2}=\sqrt{{\left({\bar{\tau }}_{1}^{\left(X^{\left(i\right)},M\right)}-{\bar{\tau }}_{1}^{\left(X^{\left(j\right)},M\right)}\right)}^{2}+\ldots +{\left({\bar{\tau }}_{F}^{\left(X^{\left(i\right)},M\right)}-{\bar{\tau }}_{F}^{\left(X^{\left(j\right)},M\right)}\right)}^{2}\;},\;i,j=\bar{1,K}.$$
  The above equation yields the symmetric matrix of Euclidean distances.
(3) Construct a dendrogram plot (UPGMA) [37] using the obtained matrix. The main goal of the dendrogram is to identify the clusters of similar time series, i.e., the clustering process involves grouping the analyzed time series based on the similarity of the slower rhythm dynamics of their synchronization with master time series M.

The procedure described above was utilized in subsequent analysis to identify the clusterization of a group of 20 people based on the synchronization of their HRV with the fluctuations in the Earth’s local magnetic field. These fluctuations are reflected by the power of the local magnetic field data. 

## 3. Results

### 3.1. The Application of the New Analysis Technique on HRV and Magnetic Field Data 

#### 3.1.1. Obtaining the Power of Local Magnetic Field during the Experiment

The local magnetic field power data was computed using the magnetic field intensity values (see Algorithm A) during the experiment (see Section 2.3.1). It is also important to note one specific feature of the acquisition process of the magnetometer data. The magnetometer values are uploaded to the central server at the end of each hour, and the time required for the upload is about one minute. Therefore, the magnetometer data contains one minute-long periods of missing data that occur at the end of each hour. 

The local magnetic field power was calculated in the frequency range ω∈[0;1] Hz, since the low-frequency fluctuations of the magnetic field have the most significant impact on human physiology, especially heart and brain activity [29].

The normal heart rate for healthy adults is approximately 60 beats per minute, which implies that the standard IBI is approximately one second. Thus, the power of the magnetic field was computed in one-second intervals, in order to match the time scales of HRV and the local magnetic field variability.

During the computation of the magnetic field power, the spectrogram was cropped using the cropping level $S_{crop}=0.25$, since empirical observations indicated that this cropping level was most effective at removing the spike type noise from the spectrograms. 

#### 3.1.2. Identification of Clusters in the Groups Based on the Similarity/Synchronization between Participants’ HRV and Magnetic Field Activity

Algorithm D was applied to the experimental data (Section 2.1 and Section 3.1.1) in order to identify clusters of participants based on the slow dynamics of the synchronization between the participants’ HRV and the power of the local magnetic field. 

According to Algorithm D, the time series $X^{\left(k\right)},\;k=\bar{1,20}$ represents the participants’ HRV data collected during the experiment. The master time series M corresponds to the time series of the power of the local magnetic field measured during the time of the experiment. 

Since Algorithm D employs Algorithms B and C, the corresponding parameters for both of those algorithms had to be selected:

(1) One of the steps of Algorithm C is splitting the participants’ HRV and local magnetic field power time series into segments. The standard length of analysis for HRV is five minutes [38]. Thus, inter-beat (RR) interval and magnetometer data was split into five-minute segments for analysis. Note that since HRV data consists of time intervals between each pair of heartbeats, the number of samples in the data vectors corresponding to each five-minute segment varies due to changes in the participants heart rate and other factors that influence HRV, such as stress and emotional states [39]. Since the power of the local magnetic field was computed for one-second time intervals, the resulting five-minute segments consisted of the same number of elements (300 data points). However, the difference in the size of the segments of HRV and the power of the local magnetic field time series did not impact the overall result of the study, since all of the segments represented the same concurrent five-minute time intervals.
(2) We selected the number of slices in Algorithm B to be 60 because it was empirically observed that a higher number would result in some empty slices.
(3) The maximal value of τ in Algorithm B was set to 50. Higher values of τ would generate too short trajectory matrices, because the five-minute segments consisted of approximately 300 elements.
(4) The value of the parameter h in Algorithm C, used for identification of slow dynamics of the synchronization between the two time series, was set to 48. This corresponded to a four-hour averaging of the difference of the optimal time lags. It was observed that this value of h produced the most meaningful averaging.
(5) As noted in Section 3.2 the magnetometer data contained one minute-long periods of missing data at the end of each hour. Since these periods in the time series did not contain any information, it was necessary to remove those periods in such a way that would not disrupt the timing between the HRV and magnetic field time series. The solution we implemented was to remove the missing data segments from both the five-minute magnetometer data and from the five-minute RR interval series. Since the cropped series obtained after this procedure fully defined the five-minute series, they were used in the data reduction step.

We applied the clusterization technique on two-day and two-week data sets collected during the experiment (see Section 3.2) in order to determine how the time span of the data set impacts the quality of the clusterization.

We firstly analyzed two days of data (2015/02/27 18:05:00 through 2015/03/01 18:05:00). Therefore, each individual’s HRV and the magnetometer data consisted of 576 five-minute segments, i.e., T=576. 

**Figure 10:**
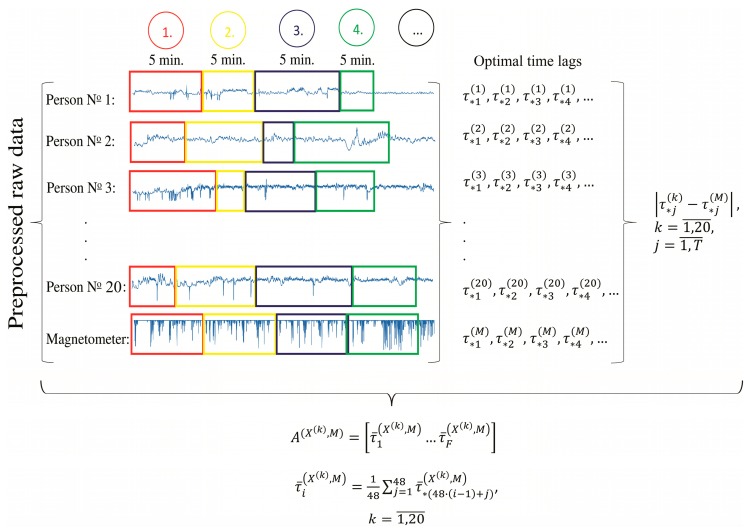
The scheme of the application of Algorithm C on the experimental data. The horizontal axis of the depicted data corresponds to the indices of the time series.

According to the first step of Algorithm D, the vector of mean absolute differences $A^{\left(X^{\left(k\right)},M\right)}$ was computed as described in Algorithm C, for each $X^{\left(k\right)},\;k=\bar{1,20}$. The execution of this step is demonstrated in Figure 10. 

**Figure 11:**
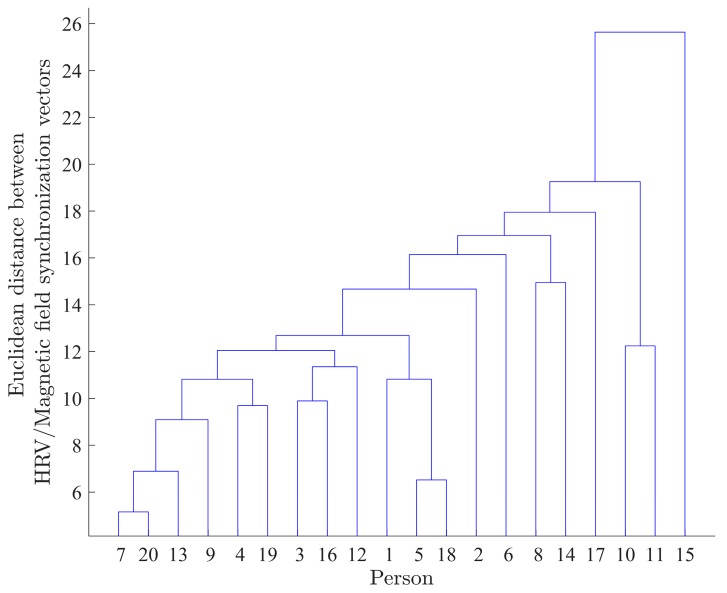
Dendrogram plot for the two-day (2015/02/27 18:05:00 through 2015/03/01 18:05:00) data. Numbers on the X axis represent participants (numbered from 1 to 20).

The application of steps 2 and 3 of Algorithm D to the two-day (2015/02/27 18:05:00 through 2015/03/01 18:05:00) data resulted in the dendrogram plot depicted in Figure 11. 

The dendrogram depicted in the Figure 11 is a visual representation of the geometrical synchronization between HRV and the magnetic field for all 20 participants. Numbers on the X axis represent participants. The height of the branches of the dendrogram is proportional to the Euclidean distance between HRV/Magnetic field synchronization vectors for corresponding participants.

It can be seen in Figure 11 that participants 7 and 20 are the closest (or most similar) in the sense of synchronization between their HRV and local magnetic field power time series. The Euclidean distance between the HRV and magnetic field synchronization for the pair of participants (7,20) is equal to 5.15. At the opposite end of the spectrum, the participant 15’s synchronization with the magnetic field is least similar to any of the remaining participants.

**Figure 12:**
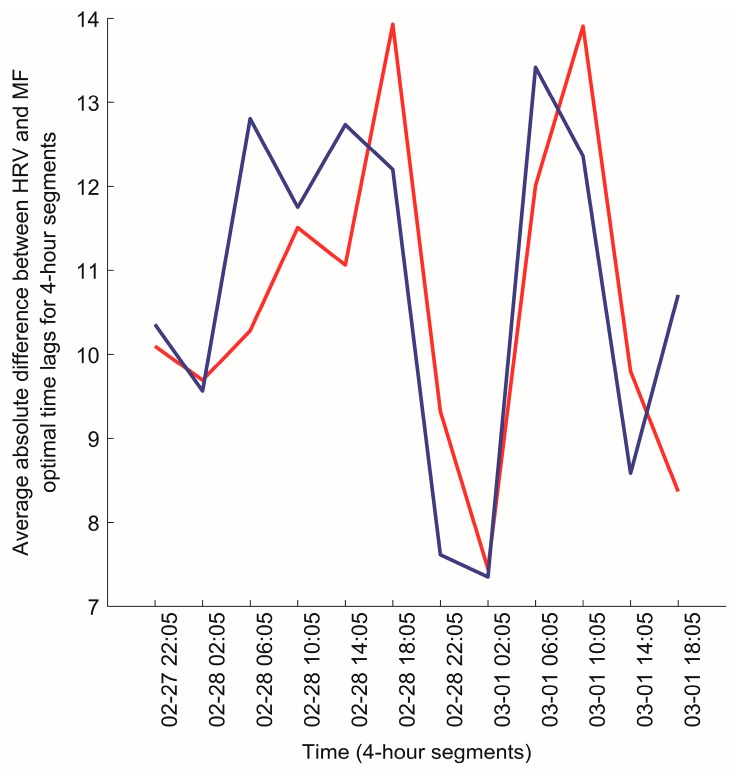
The variation of the slow dynamics of the geometrical synchronization constructed from optimal time lags for participant 7 (red line) and participant 20 (blue line) for the time period between 2015/02/27 18:05:00–2015/03/01 18:05:00.

**Figure 13:**
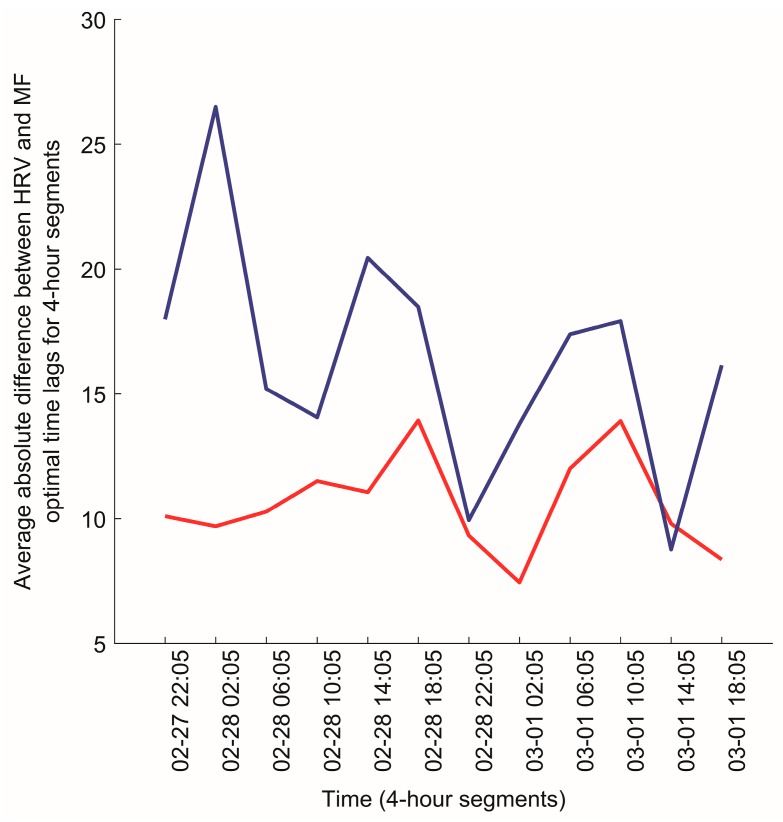
The variation of the slow dynamics of the geometrical synchronization constructed from optimal time lags for participant 7 (red line) and participant 15 (blue line) for the time period between 2015/02/27 18:05:00–2015/03/01 18:05:00.

The variation of the slow dynamics of the synchronization (Algorithm C) for two pairs of participants, (7,20) and (7,15), is also illustrated in Figure 12 and Figure 13, respectively. It can be seen that there is a strong visible similarity between the synchronization dynamics for participants 7 and 20, meaning that they are similarly synchronized with the local magnetic field, and form a cluster in the dendrogram (Figure 11). On the other hand, there is no visible similarity in the synchronization dynamics of individuals (7,15), indicating that the relationship between HRV and magnetic field activity for those participants is unlikely (Figure 13). The Euclidean distance between the HRV and magnetic field synchronization for the pair of participants (7,15) is equal to 30.09.

**Figure 14:**
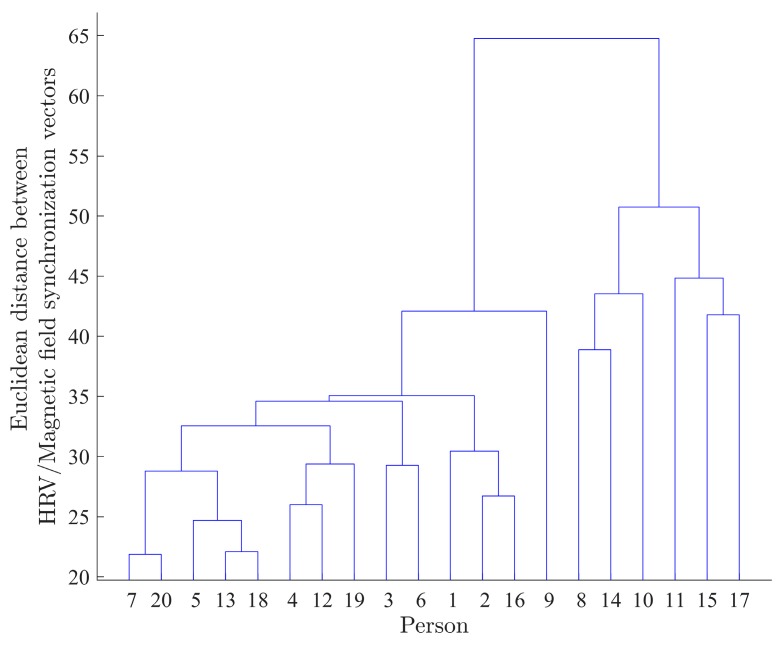
Dendrogram plot for the two-week data. Numbers on the X axis represent participants (numbered from 1 to 20).

Next, the dendrogram plot (Figure 14) for the entire two weeks (T=4032) of the experiment was obtained in an identical manner. The comparison of the two-day (Figure 11) and two-week (Figure 14) clusterization results shows that the use of the data with the longer time span provides better quality of clusterization, since the distances between the identified clusters for two-week data (Figure 14) are greater.

### 3.2. The Relation between Synchnorization Results and the Psychological Interactions between Participants

#### 3.2.1. Psychological Survey Data

In addition to the HRV data presented in Section 2.1, each person’s physical and mental condition as well as the quality of interactions between the individuals during the two-week experiment were assessed. Each participant completed a questionnaire twice each day throughout the two-week period. The questionnaire consisted of questions concerning their physical, emotional, social, and general states (rating scale between 0 and 10). At the end of each day, each participant was also asked to make a list of other participating individuals who they had interacted with that day (if any) and rate whether the interaction had positively (+1) or negatively (−1) affected them and their survey responses. The quality of interaction data is shown in Table 1. The first column as well as the first row of the table show the participant number for each of the 20 volunteers. The numbers in the intersection rows and columns equal the sum of the row person ratings of the interaction with the column person ratings over the 14 days. If, for example, the row person specified four positive and two negative interactions with the column person during the two-week experiment, the overall interaction value will equal 2. It can be seen that the matrix is nonsymmetric, which means that if the column person positively or negatively affected the row person, this does not necessarily imply that the opposite is true. The matrix is also sparse, since participants did not complete this part of the survey if interactions did not occur. 

**Figure 15:**
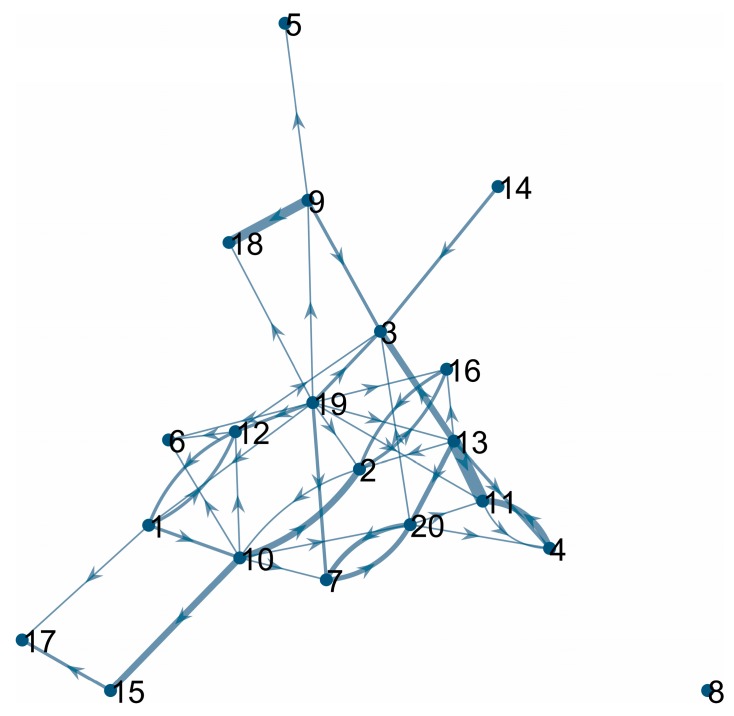
The graph of the evaluated interaction levels between participants. Nodes represent participants (numbered from 1 to 20). A line with an arrow pointing from person a to b (a,b), represents that person a feels positive about person b. The width of the line is proportional to the overall (a,b) interaction value (sum of a’s ratings of the interaction with the b’s ratings over the 14 days).

In order to illustrate interaction data, the questionnaire matrix was visualized using the directed weighted graph visualization technique (Figure 15). A line with an arrow pointing from person a to person b (the pair (a,b)) represents that person a felt positive about person b. The width of the line is proportional to the number of times such an interaction did occur. The graph gives a clearer picture of “mutual affection” between the participants. Participants’ pairs (7,20); (2,16); (4,11); (2,10); (1,12) can be clearly identified. However, it is important to note that the “mutual affection” for pairs (4,11) and (2,10) was not “balanced”, since the thickness of lines (4,11) and (11,4) as well as lines between (2,10) and (10,2) is substantially different. Consequently, only the pairs (7,20), (2,16), and (1,12) show bilateral “mutual positive interactions”.

**Figure 16:**
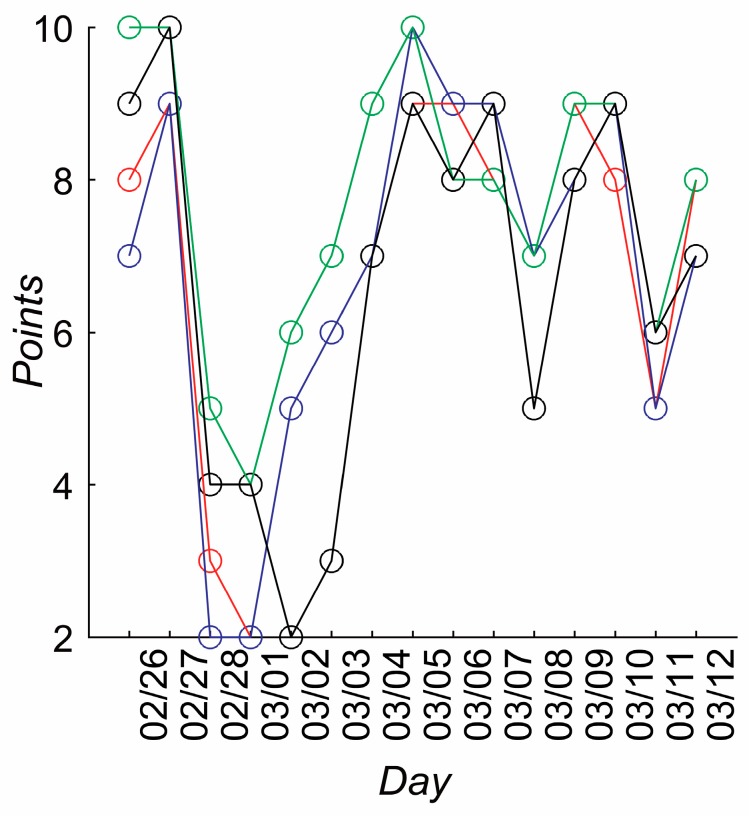
Participant 15’s change of status during self-evaluation in points (max—10, min—0; Y axis) over the 14 days (X axis). The green, black, red, and blue lines correspond to the self-evaluation of social, general, physical, and emotional states, respectively.

The data from the questionnaires (physical, emotional, social, and general state), was also analyzed and examined for the occurrences of changes in participants’ conditions in each domain. Changes were most clearly evident in participant 15’s survey data (Figure 16). The figure shows that after feeling good for the first two days of the experimental period, there was a change in his physical, emotional, social, and general condition that drastically worsened and then recovered on the eighth day of the experiment. 

#### 3.2.2. Comparison of Survey Data and the HRV/Magnetic Field Synchronization Results

The results of the HRV geometrical synchronization with the magnetic field data (represented by cluster diagrams in Section 3.1.2) were compared with the survey data (Section 3.2) in order to determine if the two separate data sets (sociological and physiological) revealed similar trends.

The dendrogram in Figure 14 shows that the synchronization between HRV and local magnetic field power for participants 7 and 20 is mostly similar. On the other hand, the synchronization for participant 15 is mostly different when compared to all other participants. 

It is interesting to observe that the pair of participants (7,20) is the mostly mutually positively oriented pair according to the questionnaire data (Figure 15). Remarkably, participant 15 has self-assessed his condition being the worst (out of all 20 participants) during the analyzed period of time. 

It appears that the computational technique based on the identification of slow dynamics of the synchronization between HRV and local magnetic field can also reflect interpersonal relationships. Participants reporting more positive states and interactions were more similarly synchronized with the magnetic field. Note that the questionnaire data was not used in the proposed algorithm, and served only as a tool to assess psychological relationships within the group.

## 4. Discussion

This study developed and validated a novel computational approach using near-optimal chaotic attractor embedding techniques for the identification of physiological synchronization among individual group members’ slow wave rhythms in heart rate variability and the degree of synchronization with changes in the local geomagnetic field. This approach allowed us to identify and quantify the degree of geometrical synchronization in time. This new analysis method was utilized to determine the degree of synchronization between locally obtained geomagnetic fields and to identify clusters of similar synchronization patterns in a group of 20 people whose HRV was continuously monitored over a two-week period as they went about their normal day-to-day lives.

Through comparing the two-day and two-week clusterization results, it can be seen that the two-week data provided better separation of the clusters of participants, i.e., the distances between the constructed clusters are greater. This demonstrates that the longer duration of the experiment positively impacts the ability to identify meaningful clusters of individuals. However, the comparison of the two-day dendrogram with the survey data showcased that a shorter time span of data provides a clearer detection of the changes in the participants’ condition. This is because the changes in the participant’s condition can average out over a long period of time. Thus, such investigations should be performed over both short and long time periods in order to obtain more complete results. 

To the best of our knowledge, this is the first study to incorporate psychological data gathered throughout the experiment in the context of physiological synchronization to other group members and with the Earth’s time-varying magnetic fields. 

Interestingly, the synchronization between the groups’ slow wave dynamics of RR intervals and the variation of the local magnetic field were consistent with the psychological data gathered throughout the experiment. When individual pairs reported more stress in their interpersonal relationships, they were less synchronized. This could imply that both the physiological and psychological variables were influenced by the time-varying magnetic fields in the environment. On the other hand, it may indicate that one’s level of stress and emotional state modulates the capacity to synchronize to other group members and the Earth’s magnetic field. Either way, this finding suggests that psychological states may be a factor in mediating the level of physiological synchronization between people and with the rhythms in the Earth’s magnetic field.

Although the specific details for how geomagnetic fields influence human psychophysiology are not yet fully understood, a potential explanation is through a resonant coupling between the nervous system and filed line resonances (Alfvén waves), or standing waves in the Earth-ionosphere resonant cavity (Schumann resonances) that overlap with physiological rhythms [30]. However, a growing body of research strongly suggests that solar and magnetic influences affect a wide range of human health and behavioral processes with the cardiovascular and nervous systems being the most clearly affected.

Overall, the study demonstrated that the slow wave rhythms in heart rate variability can synchronize with local magnetic field data, and that the degree of synchronization is affected by the quality of interpersonal relationships. When two or more persons respond to some changing environmental factor in a similar way and are emotionally close as measured by an independent metric (such as the survey or a direct comparison of their HRV attractors over time), then their response patterns to the environmental factor are less likely to result from chance. 

## 5. Conclusions

The results of this study are consistent with other studies showing that daily autonomic nervous system activity responds to changes in geomagnetic activity. It also confirms these findings in a larger group and by using a different analysis approach; i.e., the observation of slow wave dynamics occurring in people’s heart rhythms over many hours to days. In addition, it also confirms the surprising degree of synchronized activity between the slow wave dynamics in heart rhythms and changes in the Earth’s time-varying magnetic field in a frequency range that includes both Schumann resonances and geomagnetic field line resonances, which have similar frequencies as the rhythms produced by human brains and hearts.

## Figures and Tables

**Table 1:** Interpersonal interaction data, gathered from all questionnaires.

*N*	1	2	3	4	5	6	7	8	9	10	11	12	13	14	15	16	17	18	19	20
1			−1	−1						2		2					1			−1
2										1						2				
3																				
4			−1								4									
5																				
6																				
7																				3
8																				
9			2		1													6		
10		4				1	1					1			4					1
11				1																
12	2		1			1													−1	
13		1	4	2							8					1				3
14			2																	
15																	2			
16		2																		
17																				
18																				
19	1	1	2			1	2		1		1	2	1			1		1		
20			1	1			3				1									

## Supplementary Materials

The following are available online at www.mdpi.com/1660-4601/14/9/998/s1, Ethical Statement: LEIDIMAS ATLIKTI BIOMEDICINI TYRIMA.

## Abbreviations

EEG	Electroencephalogram
HRV	Heart rate variability
IBI	Inter-beat-interval
Pc	pulsations continuous
Pi	pulsations irregular
