# Supplementary material for: Identification of a Group’s Physiological Synchronization with Earth’s Magnetic Field
Source: Int J Environ Res Public Health. 2017 Sep 1;14(9):998. doi: 10.3390/ijerph14090998 (PMC5615535; doi:10.3390/ijerph14090998)
Supplement: Supplementary file 1 [file ijerph-14-00998-s001.zip › Supplemental1.pdf]

KAUNO REGIONINIS BIOMEDICININIŲ TYRIMŲ ETIKOS KOMITETAS
Lietuvos sveikatos mokslų universitetas, A. Mickevičiaus g. 9, LT 44307 Kaunas, tel. (+370) 37 32 68 89;el.paštas: kaunorbtek@lsmuni.lt

LEIDIMAS ATLIKTI BIOMEDICININĮ TYRIMĄ

2015-12-23 Nr. BE-2-51

Biomedicininio tyrimo pavadinimas: **"Psichofiziologinių žmogaus parametrų sąsajos su žemės magnetinio lauko svyravimais Lietuvoje"**	
Protokolo Nr.:	**Joffė-Vain-1**
Data:	**2015-12-16**
Versija:	**2**
Asmens informavimo forma	**Versija 2, 2015-12-16**
Pagrindinis tyrėjas:	**Prof. habil. dr. Alfonsas Vainoras**
Biomedicininio tyrimo vieta:<br>Įstaigos pavadinimas:<br>Adresas:	**Lietuvos sveikatos mokslų universitetas**<br>**Sveikatos tyrimų institutas**<br>Betonuotojų g. 4, Kaunas

Išvada:
Kauno regioninio biomedicininių tyrimų etikos komiteto posėdžio, įvykusio **2015 m. gruodžio mėn. 22 d.** (protokolo Nr. BE-10-12) sprendimu pritarta biomedicininio tyrimo vykdymui.

Mokslinio eksperimento vykdytojai įsipareigoja: (1) nedelsiant informuoti Kauno Regioninį biomedicininių Tyrimų Etikos komitetą apie visus nenumatytus atvejus, susijusius su studijos vykdymu, (2) iki sausio 15 dienos – pateikti metinį studijos vykdymo apibendrinimą bei, (3) per mėnesį po studijos užbaigimo, pateikti galutinį pranešimą apie eksperimentą.

Kauno regioninio biomedicininių tyrimų etikos komiteto nariai			
Nr.	Vardas, Pavardė	Veiklos sritis	Dalyvavo posėdyje
1.	Prof. Romaldas Mačiulaitis	Klinikinė farmakologija	taip
2.	Prof. Edgaras Stankevičius	Fiziologija, farmakologija	taip
3.	Doc. Eimantas Peičius	Filosofija	taip
4.	Dr. Ramunė Kasperavičienė	Kalbotyra	taip
5.	Med. dr. Jonas Andriuškevičius	Chirurgija	taip
6.	Agnė Krušinskaitė	Teisė	taip
7.	Prof. Skaidrius Miliauskas	Pulmonologija, vidaus ligos	taip
8.	Med. dr. Rokas Bagdonas	Chirurgija	ne
9.	Eglė Vaižgelienė	Visuomenės sveikata	ne

Kauno regioninis biomedicininių tyrimų etikos komitetas dirba vadovaudamasis etikos principais nustatytais biomedicininių tyrimų Etikos įstatyme, Helsinkio deklaracijoje, vaistų tyrinėjimo Geros klinikinės praktikos taisyklėmis.

Pirmininkas Prof. Romaldas Mačiulaitis

LIETUVOS SVEIKATOS MOKSLŲ UNIVERSITETAS * KAUNO REGIONINIS BIOMEDICININIŲ TYRIMŲ ETIKOS KOMITETAS
