# Supplementary material for: Identification of a Group’s Physiological Synchronization with Earth’s Magnetic Field
Source: Int J Environ Res Public Health. 2017 Sep 1;14(9):998. doi: 10.3390/ijerph14090998 (PMC5615535; doi:10.3390/ijerph14090998)
Supplement: Supplementary file 1 [file ijerph-14-00998-s001.zip › Supplemental2.pdf]

LR Asmens duomenų teisinės apsaugos įstatymo 10 str. 3 punktas numato, jog asmens duomenys apie asmens sveikatą automatiniu būdu, taip pat mokslinio **medicininio tyrimo tikslais** gali būti tvarkomi tik pranešus Valstybinei duomenų apsaugos inspekcijai. Šiuo atveju Valstybinė duomenų apsaugos inspekcija privalo atlikti išankstinę patikrą.

**Pasibaigus tyrimui, tyrėjas ar tyrimo užsakovas privalo informuoti KRBTEK raštu apie tyrimo pabaigą bei pateikti tyrimo ataskaitos santrauką. Atliekant biomedicininio tyrimo pakeitimus būtina gauti Kauno regioninio biomedicininio tyrimo etikos komiteto pritarimą pakeitimams.**

Reikalavimas pateikti pranešimą apie tyrimo pabaigą bei ataskaitos santrauką įsigaliojo nuo 2010 m. gegužės 6 d. Šį reikalavimą rasite Lietuvos Respublikos sveikatos apsaugos ministro įsakymo "Dėl leidimų atlikti biomedicininį tyrimą išdavimo tvarkos aprašo patvirtinimo" (Žin., 2008, Nr. 6-225; 2010, Nr. 55-2706; 2011, Nr. 233-1570; Nr. 67-3184) $18^1$ punkte *„Leidimas atlikti biomedicininį tyrimą galioja iki biomedicininio tyrimo paraiškoje nurodytos tyrimo pabaigos datos. Biomedicininių tyrimų užsakovas, jo įgaliotas atstovas ir (ar) pagrindinis tyrėjas per 30 kalendorinių dienų privalo raštu pranešti leidimą atlikti biomedicininį tyrimą išdavusiai institucijai (Lietuvos bioetikos komitetui ar regioniniam biomedicininių tyrimų etikos komitetui) apie tyrimo pabaigą ir per 90 kalendorinių dienų pateikti tyrimo vykdymo ataskaitos santrauką"*.

Įsakymo nuostata taikoma visiems biomedicininiams tyrimams.
